# Simultaneous detection of decidual Th1/Th2 and NK1/NK2 immunophenotyping in unknown recurrent miscarriage using 8-color flow cytometry with FSC/Vt extended strategy

**DOI:** 10.1042/BSR20170150

**Published:** 2017-05-19

**Authors:** Peng Dong, Xi Wen, Jia Liu, Cui-Yan Yan, Jing Yuan, Lan-Rong Luo, Qiao-Fei Hu, Jian Li

**Affiliations:** 1 Department of Obstetrics and Gynecology, Xuanwu Hospital, Capital Medical University, Beijing, China; 2 Family Planning Department, Beijing Obstetrics and Gynecology Hospital, Capital Medical University, Beijing, China

**Keywords:** Unexplained recurrent miscarriage, multiparametric flow cytometry, 8-color, Th1/Th2, NK1/NK2, FSC/Vt

## Abstract

Th1/Th2 imbalance is considered as a mechanism for recurrent miscarriage. The NK1/NK2 paradigm is hypothesised to play an important role in pregnancy. However, few results showed simultaneous changes of these subsets *in vivo* in decidual tissues. The present study aimed to detect the decidual mononuclear cells (dMo), and the Th1/Th2, and NK1/NK2 paradigm simultaneously using multiparametric flow cytometry (MFC) in unexplained recurrent miscarriages (URM). Mononuclear cells were isolated from the decidual tissues of URM cases and early pregnant women. The mononuclear cell percent was demonstrated by detecting the expression of CD3, CD4, CD8, CD56, and CD16 extracellular markers, interferon (IFN)-γ, and interleukin (IL)-4 intracellular markers in live cells using 8-color flow cytometry with forward scatter (FSC)/side scatter (SSC) and FSC/viability (Vt) initial gating strategies, and the ratios of Th1/Th2 and decidual NK1 (dNK1)/decidual NK2 (dNK2) cells were compared between the subject groups. Two initial gating strategies of the FSC/SSC or FSC/Vt, with central or extended gating scales, were adapted, and there was no main effect or interaction for the cell proportions, except for the type 1 and type 2 subsets in the FSC/Vt extended gating strategy. There was no significant difference of the proportions of the decidual T, dNK, NKT-like, Th, and Tc cells between the two groups. However, the Th1/Th2 and dNK1/dNK2 ratios in the URM patients were higher compared with the normal group when using the FSC/Vt extended gating strategy. The present study provides means to detect Th1/Th2 and dNK1/dNK2 simultaneously in URM patients for large sample investigations in the future.

## Introduction

Spontaneous miscarriage is the most common complication of early pregnancy. Recurrent spontaneous miscarriage (RM), defined by two or more failed pregnancies, occurs in 1–2% of fertile women [1,2]. Present knowledge about the etiology of RM mainly includes parental or fetal chromosomal defects, anatomical anomalies, endocrine disorders, infections, and maternal autoimmune factors [3,4]. A considerable number of trials targetting the feto–maternal interface have been carried out for decades, including theoretical models covering alloimmunity, autoimmunity, and the innate immune system. Both a T helper (Th) cell imbalance and a natural killer (NK) cell-mediated immunity contribute to the unknown mechanisms underlying unexplained recurrent miscarriage (URM) cases [5–8].

The concentration of Th2 cytokines is higher in normal pregnancy, and Th1 cytokines are dominantly produced in the URM patients, either in the peripheral blood, the secretory endometrium, or the decidua, indicating a distinct Th2-bias in normal pregnancy and a Th1-bias in URM [9–13]. These are not the only cells possessing type 1/2 immune responses. Type-1 and type-2 cytokines secreted by NK cells, which play important roles in autoimmunity, cancer, transplantation, and pregnancy [14], also have been investigated [15]. NK1/NK2 and NKT1/NKT2 cell ratios were significantly decreased in normal early pregnancy compared with non-pregnancy levels [16]. The NK1 percentage in peripheral blood NK cells is shifted in RM [17,18], and although they are rare in the decidua, these are slightly elevated in miscarriage [19].

Although many studies have examined the Th1/Th2 and NK1/NK2 paradigms separately, there are less results showing the simultaneous changes of these subsets *in vivo* in the decidual tissues. Moreover, networks between these type 1/2 lineages and their parental lineages in URM have not been elucidated. Discovering a comprehensive phenotype in the feto–maternal interface is urgent in URM and is useful when using a larger number of sample investigations to identify the role of a comprehensive phenotype in URM diagnosis in the future.

Fortunately, the development of flow cytometers and fluorochromes has enabled researchers to explore the multitude of immune system subsets, which has led to the establishment of polychromatic or multiparametric flow cytometry (MFC). With MFC, we can analyze optical protocols with 11–17 parameters in a single run [20–22]. Decidual mononuclear cells (dMo) have been distinguished using flow cytometers with forward scatter (FSC)/side scatter (SSC) as an initial gating in peripheral blood [23]. In the present study, we wanted to define the decidual immune cells and to simultaneously detect changes in the Th1/Th2 and decidual NK1 (dNK1)/decidual NK2 (dNK2) ratios of the decidual tissues using flow cytometery. However, gating on the physical parameters of permeabilised cells may be of limited use because the cells tend to shrink, and a number of positive events may be gated out because activated cells may have a larger scattering than resting ones [24]. Thus, we used 8-color flow cytometry with two gating strategies, including FSC/SSC and FSC/Vt, to determine whether this powerful counting technique could be used to study decidual immune cells and to simultaneously detect changes in the Th1/Th2 and dNK1/dNK2 ratios *in vivo* in the decidual tissues.

## Materials and methods

### Study participants and sampling

We performed the present study at the Beijing Obstetrics and Gynecology Hospital in China. Ethical approval was obtained for the present study. Placental tissues were harvested after obtaining informed consent from participants with a periodical menstrual circle. URM cases (6–11 weeks of gestation, *n*=20) were defined as having a history of two or more consecutive abortions and no pregnancy-related infections, uterus abnormalities, parental or fetal chromosomal abnormalities, endocrine disorders, congenital thrombophilia, and autoimmune diseases. We defined the period of the gestational age based on the last menstruation. None of the male partners of these cases had any infertility issues. Dilation and evacuation were performed within 3 days after the diagnosis of fetal loss under ultrasound. For the normal pregnancy women (6–11 weeks of gestation, *n*=20), elective abortions were performed within 1 week after the fetal heart activities were identified. Villous and decidual tissue were placed into two sterile samplers with RPMI-1640 medium supplemented with 10% FBS, 2% L-glutamine (Gibco, Carlsbad, CA, U.S.A.), and 1% penicillin-streptomycin, and the samples were transferred to the laboratory within 1 h in an ice box. The villous tissue was sent for genetic analysis using G-banding karyotyping. The decidua was finely minced to almost 1 × 1 × 1 mm pieces for the following steps.

### Isolation of dMo

The minced tissue was mechanically ground and filtered, and the cells were then isolated by the density gradient method [23]. Cells with a concentration of 0.5–1 × 10^6^/ml were stimulated according to the technique originally described by Jung et al. [25] and Picker et al. [26], with a leukocyte activation cocktail (BD Pharmingen, BD Biosciences, San Jose, CA, U.S.A.), which is a combination containing the PMA, calcium ionophore (ionomycin), and the protein transport inhibitor Brefeldin A (BFA). The cells were cultured for 4 h in a 37°C humidified CO_2_ incubator in the medium to be stimulated. After harvesting, the cells were washed with cold staining buffer (BD Pharmingen, BD Biosciences, San Jose, CA, U.S.A.) for use in the antibody staining protocols.

### Antibody staining, fixation, and permeabilisation

Fluorescent-coupled monoclonal antibodies were utilised to analyze the surface (CD3, CD4, CD8, CD56, and CD16) and intracellular antigens (interferon (IFN)-γ and interleukin (IL)-4) (Table 1). Fixable viability dye (FVD) eFluor 506 (eBioscience, San Diego, CA, U.S.A.) was used to label dead cells prior to fixation. For immunofluorescent staining, 100 μl of stimulated cell suspension was stained with the surface antibodies in the dark at room temperature for 20 min and was then fixed and permeabilised with an Intracellular Fixation & Permeabilization Buffer Set (eBioscience, San Diego, CA, U.S.A.) according to the manufacturer’s instructions. After permeabilisation, the cell suspension was incubated with intracellular antibodies in the dark at room temperature for 20 min. The cells were washed twice with cold staining buffer and were then loaded for data collection. An isotype control was used for the CD16 antibody, and a fluorescence minus one (FMO) control was used for the IFN-γ and IL-4 antibodies.

**Table 1:** Antibody panel used in the present study

Antibody	Fluorochrome	Clone	Isotype	Source
CD3	PerCP	BW264/56		Miltenyi
CD4	APC	M-T466		Miltenyi
CD8	FITC	BW135/80		Miltenyi
CD56 (N-CAM)	PE-Vio770	REA196		Miltenyi
CD16	VioBlue	VEP13	Mouse-IgM	Miltenyi
IFN-γ	APC-Vio770	45-15		Miltenyi
IL-4	PE	7A3-3		Miltenyi
Mouse-IgM	VioBlue	IS5-20C4		Miltenyi

### Flow cytometry data collection

Each sample was loaded and recorded with a BD FACSCanto Plus flow cytometer (BD Biosciences, San Jose, CA, U.S.A.). The cytometer performance was checked prior to each experiment. Photomultiplier tubes (PMTs) collected the fluorescent light activated by a solid-state laser of 405nm (FVD eFluor 506, VioBlue), 488 nm (FITC, PE, PerCP, PE-Vio770), and 640nm (APC, APC-Vio770). The PMT voltages were adjusted using the cell sample and were permanent in all experiments. The fluorescence compensation was performed using a single-antibody-labeled COMPtrol Goat anti-Mouse Ig (H&L) Particle Kit (Spherotech, Lake Forest, IL, U.S.A.) and then with the single-antibody-labeled cell samples. For each sample analyzed, 150000 events were collected.

### Data analysis

The initial gating was applied with FSC/Vt gating methods, compared with FSC/ SSC regular gating methods. Adhesion events were excluded. Live cells from the FSC/Vt gates or the Vt/histogram were gated with the CD3/CD56 quadrant gate. The CD4/CD8 quadrant gate was set within the CD3^+^CD56^–^ subsets, and the CD56^bright^CD16^–^ cells were identified within the CD3^–^CD56^+^ subsets. The NKT-like cells were identified as the CD3^+^CD56^+^ subsets. The quadrant gates of IFN-γ/IL-4 were set for the Th (CD3^+^CD4^+^), Tc (CD3^+^CD8^+^), dNK (CD3^–^CD56^bright^CD16^−^), and NKT-like lymphocytes (CD3^+^CD56^+^). Each subpopulation was quantified as a percentage of the parental population. Based on these two methods, centralised and extended scales were attempted. The initial centralised gate was set on the brightest part of a pseudocolor plot. The initial extended gate was set to include the peripheral of the brightest part. Generally, FSC/SSC (central), FSC/SSC (extended), FSC/Vt (central), and FSC/Vt (extended) gates were applied. All of the flow data were processed with FlowJo software 10.0 (TreeStar, Ashland, OR, U.S.A.).

### Statistical analyses

The statistical analyses were performed using SPSS Statistics software 22.0 (IBM, Armonk, NY, U.S.A.). The age and cell percentage data are presented as the mean ± S.E.M. (M ± S.E.M.). A two-way ANOVA under a significance level of 0.05 for factorial design was applied to compare the effects of the four gating strategies. For two independent samples, a *t* test at a two-tailed significant level of 0.05 was performed for comparison of the cell percentages between the two subject groups.

## Results

The clinical characteristics of women with URM and normal pregnancies are summarised in Table 2. URM patients with two to five miscarriages had a mean age of 27.1 ± 3.5 (range: 24–34) years, with a mean gestational age of 63.1 ± 8.1 days. The normal pregnancy women with at least one living child and no history of spontaneous miscarriage, had a mean age of 28.2 ± 5.8 (range: 23–32) years, with a mean gestational age of 58.1 ± 8.7 days.

**Table 2:** Clinical characteristics of women with URM and normal pregnancy

	URM	Normal pregnancy	*P* (*t* test)
Age	27.1 ± 3.5	28.2 ± 5.8	NS*
Gestational age (days)	63.1 ± 8.1	58.1 ± 8.7	NS
Gravidity	2.8 ± 1.1	3.1 ± 0.7	NS
Parity	0	2.8 ± 0.5	*P*<0.001
URMs	2.8 ± 1.1	0	*P*<0.001

*NS, not significant.

To exclude the impact of infectious diseases, genital system infections were routinely monitored for both groups, including bacterial vaginosis, candidiasis, trichomonas vaginitis, Bartholin’s duct cyst and abscess, chronic pelvic infection, and cervicitis. Moreover, any sign of bacteremia or viremia was monitored before surgery, such as fever, shivering, and neutrophilia. An antiphospholipid antibody test and an antinuclear antibody test were performed to exclude the impact of autoimmune diseases, including antiphospholipid syndrome, systemic lupus erythematosus, Sjögren’s syndrome, rheumatic arthritis, scleroderma, mixed connective tissue disease, polymyositis, dermatomyositis, autoimmune hepatitis, and drug-induced lupus.

After the mechanical separation step, the mean wet weight of the samples was 13.5 ± 0.4 g. The concentration of the filtered cell suspension was 1.5 ± 0.6 × 10^8^/ml, which was always between 1–2 × 10^6^/ml in the isolated mononuclear cell suspension. At the end of the isolation procedure, the viability (Vt) was still greater than 90%.

**Figure 1:**
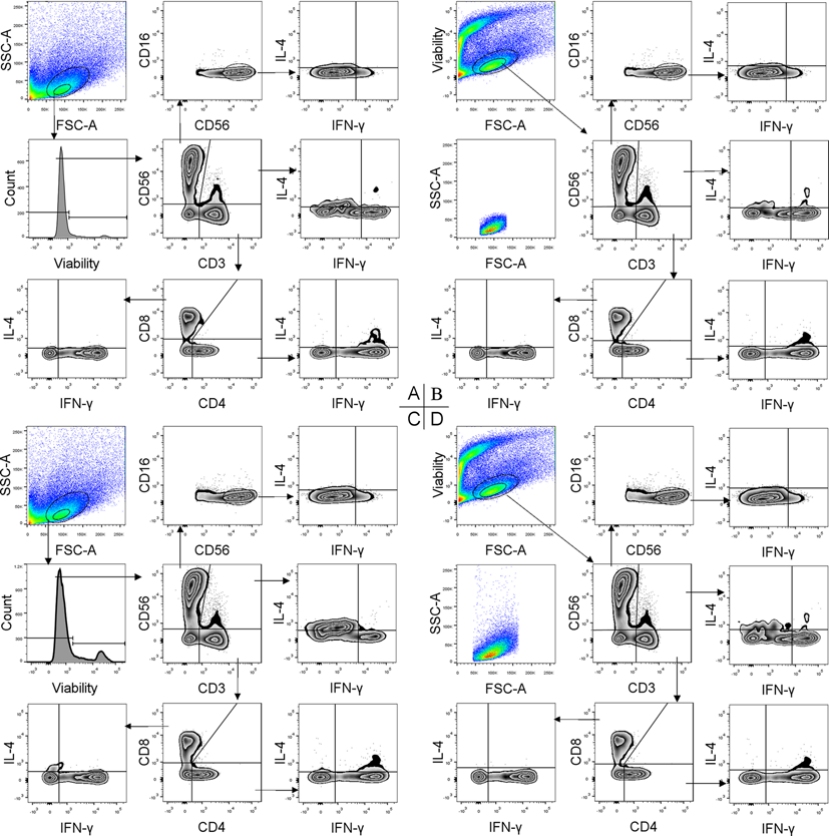
Four strategies for gating dMo in 8-color MFC The cells were stained for CD3, CD56, CD16, CD4, CD8, IFN-γ, and IL-4 with a Vt dye. The dMo are shown separately by an FSC/SSC gate (**A**) and an FSC/Vt gate (**B**) with a central scale or by an FSC/SSC gate (**C**), and an FSC/Vt gate (**D**) with an extended scale. The next gating steps were the same for each strategy except a bisector gate was added for the events obtained from the FSC/SSC gate (A,C) to exclude the dead cells. Decidual T, NK, NKT-like, Th and Tc cells and their type-1 or type-2 subsets are delineated by all these strategies. The events obtained from the FSC/Vt gate were reviewed by the FSC/SSC gate with both scales (B,D), which showed that there were more events in the FSC/Vt gate than in the FSC/SSC gate, especially with the extended scale.

**Figure 2:**
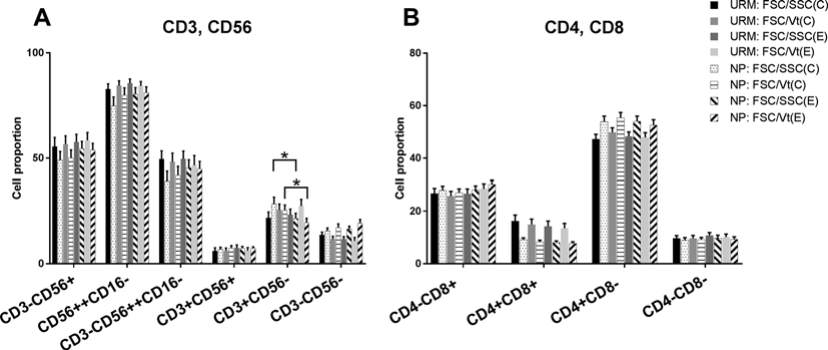
Cell percent counted by the four gating strategies (**A**) The cell percent of decidual T, NK, and NKT-like cells in the URM group and the normal pregnant group using four gating strategies. (**B**) Represents the percent of Th and Tc cells in the URM group and the normal pregnancy group using the four gating strategies. There was no statistical difference in the proportions of the dNK and NKT-like cells and the Th and Tc subsets when using the different gating strategies. The asterisk represents a statistically significant difference with *P*<0.05.

Four gating strategies were applied to analyze both the URM and control groups. According to these strategies, the CD3^+^CD4^+^CD8^−^ Th, CD3^+^CD4^–^CD8^+^ Tc, CD3^+^CD56^+^ NKT-like, and CD3^–^CD56^bight^CD16^−^ dNK plots and their type 1 or type 2 subpopulations were all visualised explicitly (Figure 1). The percentages of each subpopulation in the two groups under each strategy are presented in Tables 3 and 4. A two-way ANOVA for factorial design indicated that neither the main effect of the method nor that of the scale was statistically significant, and no statistically significant interaction between these two factors was found for any of the cell subsets no matter which group (*P*>0.05) (Figure 2).

**Table 3:** Subpopulation proportions delineated by FSC/SSC (central), FSC/SSC (extended) strategies in normal pregnancy and URM

Marker	Cell	FSC/SSC (central)		FSC/SSC (extend)	
		Normal pregnancy	URM	Normal pregnancy	URM
CD3^+^CD56^–^	T	28.4 ± 3.2	27.5 ± 3.1	21.5 ± 2.4	23.3 ± 2.7
CD3^–^CD56^bright^CD16^–^	dNK	39.2 ± 4.6	46.6 ± 4.6	45.4 ± 3.7	49.6 ± 3.7
CD3^–^CD56^bright^CD16^–^/CD3^+^CD56^–^	dNK/T	1.5 ± 0.5	1.8 ± 0.2	2 ± 0.2	2.0 ± 0.2
CD3^+^CD56^+^	NKT-like	6.9 ± 0.9	6.2 ± 1.5	7.5 ± 0.7	7.4 ± 1.5
CD3^+^CD4^+^CD8^–^	Th	53.9 ± 2.1	47.9 ± 1.9	54.0 ± 2.0	48.3 ± 1.7
CD3^+^CD4^–^CD8^+^	Tc	27.9 ± 1.6	28.5 ± 1.9	28.1 ± 1.5	26.7 ± 1.7
CD3^+^CD4^+^CD8^–^/CD3^+^CD4^–^CD8^+^	Th/Tc	2 ± 0.2	1.8 ± 0.2	2.1 ± 0.1	1.9 ± 0.2
CD3^+^CD4^+^CD8^–^IFN-γ^+^IL-4^–^	Th1	37.4 ± 2.5	43.9 ± 1.8	34.8 ± 1.9	42.7 ± 1.7
CD3^+^CD4^+^CD8^–^IFN-γ^–^IL-4^+^	Th2	1.2 ± 0.3	0.4 ± 0.1	0.8 ± 0.2	0.9 ± 0.2
CD3^+^CD4^+^CD8^–^IFN-γ^+^IL-4^+^	Th DP*	4.8±0.7	3.8 ± 0.9	3.2 ± 0.4	5.6 ± 1.1
CD3^+^CD4^+^CD8^–^IFN-γ^+^IL-4^–^ /CD3^+^CD4^+^CD8^–^IFN-γ^–^IL-4^+^	Th1/Th2	74.3 ± 14.6	167.0 ± 11.5	103.4 ± 23.0	82.5 ± 15.2
CD3^+^CD4^–^CD8^+^IFN-γ^+^IL-4^–^	Tc1	49.5 ± 2.8	57.1±3.6	43.7±2.4	56.8 ± 2.9
CD3^+^CD4^–^CD8+IFN-γ^–^IL-4^+^	Tc2	1.4 ± 0.3	2.0±1.4	3.9±0.9	2.1 ± 0.8
CD3^+^CD4^–^CD8+IFN-γ^+^IL-4^+^	Tc DP*	2.9 ± 0.5	1.6 ± 0.4	3.3 ± 0.5	3.1 ± 0.6
CD3^+^CD4^−^CD8+IFN-γ^+^IL-4^−^/CD3^+^CD4^–^ CD8+IFN-γ^–^IL-4^+^	Tc1/Tc2	83.4 ± 18.5	113.7 ± 11.9	28.0 ± 5.6	39.4 ± 7.8
CD3^–^CD56^bright^CD16^–^IFN-γ^+^IL-4^–^	dNK1	9.2 ± 1.3	8.1 ± 2.0	5.2 ± 1.1	9.9 ± 2.2
CD3^–^CD56^bright^CD16^–^IFN-γ^–^IL-4^+^	dNk2	3.2 ± 0.7	0.7 ± 0.2	8.4 ± 1.4	4.0 ± 0.7
CD3^–^CD56^bright^CD16^–^IFN-γ^+^IL-4^+^	dNK DP*	0.2 ± 0.1	0.01 ± 0.006	0.3 ± 0.1	0.3 ± 0.1
CD3^–^CD56^bright^CD16^–^IFN-γ^+^IL-4^ –^ /CD3^–^CD56^bright^CD16^–^IFN-γ^–^IL-4^+^	dNk1/dNK2	11.2 ± 3.2	72.1 ± 12.1	3.5 ± 1.6	5.7 ± 2.8

*DP represents double positive cell.

**Table 4:** Subpopulation proportions delineated by FSC/Vt (central) and FSC/Vt (extended) strategies in normal pregnancy and URM

Marker	Cell	FSC/Vt (central)		FSC/Vt(extend)	
		Normal pregnancy	URM	Normal pregnancy	URM
CD3^+^CD56^–^	T	25.4 ± 2.6	25.5 ± 2.9	19.5 ± 2.1	21.8 ± 2.8
CD3^–^CD56^bright^CD16^–^	dNK	41.9 ± 4.2	48.3 ± 4.0	45.0 ± 3.4	49.6 ± 3.8
CD3^–^CD56^bright^CD16^–^/ CD3^+^CD56^–^	dNK/T	1.5 ± 0.1	2.0 ± 0.4	2.2 ± 0.1	2.2 ± 0.1
CD3^+^CD56^+^	NKT-like	7.4 ± 1.1	6.1 ± 1.4	7.3 ± 0.7	6.1 ± 1.5
CD3^+^CD4^+^CD8^–^	Th	55.5 ± 2.0	49.8 ± 5.9	52.6 ± 2.0	47.3 ± 5.9
CD3^+^CD4^–^CD8^+^	Tc	27.0 ± 1.5	25.7 ± 1.9	30.2±1.5	26.7 ± 1.9
CD3^+^CD4^+^CD8^–^/ CD3^+^CD4^–^CD8^+^	Th/Tc	2.1 ± 0.1	2.1 ± 0.2	2 ± 0.2	1.9 ± 0.2
CD3^+^CD4^+^CD8^–^IFN-γ^+^IL-4^–^	Th1	38.5 ± 2.5	46.8 ± 1.8	35.4 ± 1.9	46.8 ± 1.8
CD3^+^CD4^+^CD8^–^IFN-γ^–^IL-4^+^	Th2	0.6 ± 0.1	0.5 ± 0.1	0.5 ± 0.1	1.1 ± 0.3
CD3^+^CD4^+^CD8^–^IFN-γ^+^IL-4^+^	Th DP*	2.9 ± 0.5	4.5 ± 1.0	2.2 ± 0.3	6.6 ± 1.2
CD3^+^CD4^+^CD8^–^IFN-γ^+^IL-4^–^ /CD3^+^CD4^+^CD8^–^IFN-γ^–^IL-4^+^	Th1/Th2	111.8 ± 25.9	133.9 ± 14.7	118.3 ± 25.7	75.5 ± 15.8
CD3^+^CD4^–^CD8^+^IFN-γ^+^IL-4^–^	Tc1	50.1 ± 2.5	63.5 ± 2.4	44.0 ± 2.6	63.9 ± 2.7
CD3^+^CD4^–^CD8+IFN-γ^–^IL-4^+^	Tc2	1.5 ± 0.4	0.9 ± 0.3	3.0 ± 0.9	0.9 ± 0.3
CD3^+^CD4^–^CD8+IFN-γ^+^IL-4^+^	Tc DP*	2.4 ± 0.6	2.7 ± 0.6	3.0 ± 0.9	3.3 ± 0.7
CD3^+^CD4^−^CD8+IFN-γ^+^IL-4^−^ /CD3^+^CD4^–^ CD8+IFN-γ^–^IL-4^+^	Tc1/Tc2	134.7 ± 32.4	89.2 ± 9.2	88.9 ±3 7.6	87.0 ± 10.1
CD3^–^CD56^bright^CD16^–^IFN-γ^+^IL-4^–^	dNK1	4.3 ± 0.9	9.5 ± 2.5	3.1 ± 0.7	13.0 ± 2.9
CD3^–^CD56^bright^CD16^–^IFN-γ^–^IL-4^+^	dNk2	0.7±0.2	1.3±0.4	0.2±0.1	4.7 ± 1.7
CD3^–^CD56^bright^CD16^–^IFN-γ^+^IL-4^+^	dNK DP*	0	0.1±0.02	0	0.6 ± 0.2
CD3^–^CD56^bright^CD16^–^IFN-γ^+^IL-4^–^ /CD3^–^CD56^bright^CD16^–^IFN-γ^–^IL-4^+^	dNk1/dNK2	25.4 ± 11.4	24.1 ± 13.0	32.0 ± 10.1	7.1 ± 2.4

*DP represents double positive cell.

Two independent *t* tests indicated that there were no significant differences in the proportions of the decidual T, NK, and NKT-like cells (Figure 2A) and the Th and Tc cells (Figure 2B) between the URM and normal pregnancy groups (*P*>0.05). The dNK cells accounted for 46.6–49.6% of the URM group and 39.2–45.4% of the control group under the four analysis strategies, while T cells accounted for 21.8–27.5% of the URM group and 19.5–28.4% of the control group. The ratio of dNK/T varied from 1.8–2.2 in RM group and 1.5–2.2 in control group within the four strategies. There were no significant differences for the dNK/T ratio between the two groups (*P*>0.05). There were 47.3–49.8% Th cells in the URM group and 52.6–55.5% in the controls, while there were 25.7–28.5% Tc cells in the URM group and 27.0–30.2% in the controls, according to the different strategies. Moreover, the ratio of Th/Tc varied from 1.8–2.1 in the URM group and was 2.0–2.1 in the controls, which was not significantly different (*P*>0.05). However, in the normal pregnancy group, we found that there is a significant difference in the T-cell proportion using the FSC/SSC (central) gating strategy compared with FSC/SSC (extended) gating strategy, and there is a similar phenomenon when using the FSC/Vt (central) gating strategy compared with the FSC/Vt (extended).

**Figure 3:**
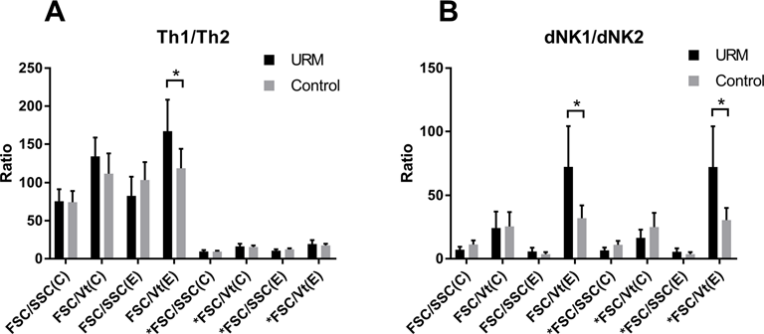
The type 1/type 2 ratio of the Th and dNK cells using the four gating strategies (**A**) The Th1/Th2 ratio in the URM group and the normal pregnancy group using the four gating strategies. There was a significant difference in the Th1/Th2 ratio in the URM group and the normal pregnant group with the FSC/Vt gating strategy. *FSC/SSC (C), *FSC/SSC (E), *FSC/Vt (C), and *FSC/Vt represent that we added the percent of the IFN-γ + IL-4 + double positive cells to both the IFN-γ + IL-4–Th1 and IFN-γ–IL-4 + Th2 percents. (**B**) The dNK1/dNK2 ratio in the URM group and the normal pregnancy group using the four gating strategies. The asterisk represents a statistically significant difference with *P*<0.05. *FSC/SSC (C), *FSC/SSC (E), *FSC/Vt (C), and *FSC/Vt represent that we added the percent of the double positive cells to both the dNK1 and dNK2 percents.

The type 1 and type 2 subpopulations of Th and dNK cells were analyzed with the four strategies separately. The ratios of the type 1/type 2 Th and dNK cells with the different scales were calculated and compared. Only with the FSC/Vt extended gating strategy were the Th1/Th2 and dNK1/dNK2 ratios in the URM group significantly higher compared with the normal group (*P*<0.05) (Figure 3).

However, there were many double positive cells in the Th subsets, accounting for 3.8–6.6% in the URM group and 2.2–4.8% in the controls. When adding the percent of the IFN-γ^+^IL-4^+^ double positive cells to both the IFN-γ^+^IL-4^−^Th1 and IFN-γ^–^IL-4^+^Th2 percents, the Th1/Th2 ratio decreased significantly with no matter which strategy was used, and there was no difference between the two groups in this situation (*P*>0.05) (Figure 3A). The number of double positives in the dNK cells was small and varied from 0.01–0.6% in the URM group and was 0–0.3% in the controls. When adding the double positive cells to the dNK1 and dNK2 percents, the dNK1/dNK2 ratio of the URM group was also significantly higher than the normal group (*P*<0.05) (Figure 3B).

## Discussion

Since Medawar and colleagues introduced the concept of immunological tolerance between a mother and her fetus [27], and Billingham theorised the breakdown of this tolerance underlying the pregnancy loss [28], a number of studies have investigated the roles of specific immune cells and molecules in the etiology of RM. For example, abortion-prone women who proceed to have a successful pregnancy are more Th2 biased than abortion-prone women who abort, and recurrent aborters who undergo a spontaneous abortion have a stronger Th1 bias than aborters who have normal pregnancy [10]. NK-1 cytokines are important for maintaining pregnancy, and the cytokine production of peripheral blood NK cells is dysregulated in women with a history of reproductive failure [29]. Previously, we observed a significantly increased NKT-like cell percentage (CD3^+^CD56^+^ and CD3^+^CD56^+^CD16^+^) in the peripheral blood and decidua of RM women than that of normal pregnant and non-pregnant women [13].

The simultaneous analysis of NKT-like and NK subpopulations inspired us to explore MFC for an analysis of the constitution of decidual immune cells. In the present study, we used eight fluorochromes with FSC/SSC (central), FSC/SSC (extended), FSC/Vt (central), and FSC/Vt (extended) gating strategies to delineate the surface markers CD3, CD4, CD8, CD56, and CD16 and the intracellular cytokines IFN-γ and IL-4 of ddMo, including a Vt marker. Using this 8-color flow cytometry, the Th, Tc, NK, and NKT-like cell populations and the Th1/Th2 and dNK1/dNK2 ratios were assessed.

According to the FSC/Vt extended analysis strategy, there was a significant increase in Th1 and dNK1 cells in the URM group compared with the control group. This is consistent with other studies and indicates an inflammatory immune reaction underlying the mechanism of URM. Although there have been plenty of studies focussing on this type 1/type 2 shift phenomenon in Th cells and dNK cells, the present study showed an overall decidual Th1/Th2 and dNK1/dNK2 profile that validated the Th and dNK type1/type2 shift simultaneously for the first time.

This 8-color analysis in a single-tube method that permits an overall assessment and is advantageous because of the fact that fewer samples, processes and reagents are needed. The FSC/SSC gate is traditionally adopted for the immunophenotyping of peripheral blood. dMo are distinguished in the dot plot by the FSC/SSC as in peripheral blood [23]. However, gating on the physical parameters of permeabilised cells may be of limited use because cells tend to shrink, and a number of positive events may be gated out because activated cells may have a larger scatter than resting ones [24]. Therefore, in the present study, we employed a Vt parameter as an initial gate to avoid the influence of the possible alteration of the SSC parameter in permeabilised and activated cells, but the FSC cannot be abandoned because it is used for the exclusion of debris. Therefore, the two scales are considered an alternate of the FSC parameter. The 2 × 2 factorial design showed that there were no main or interaction effects regarding the composition of the dMo with or without an SSC and with different FSC scales.

**Figure 4:**
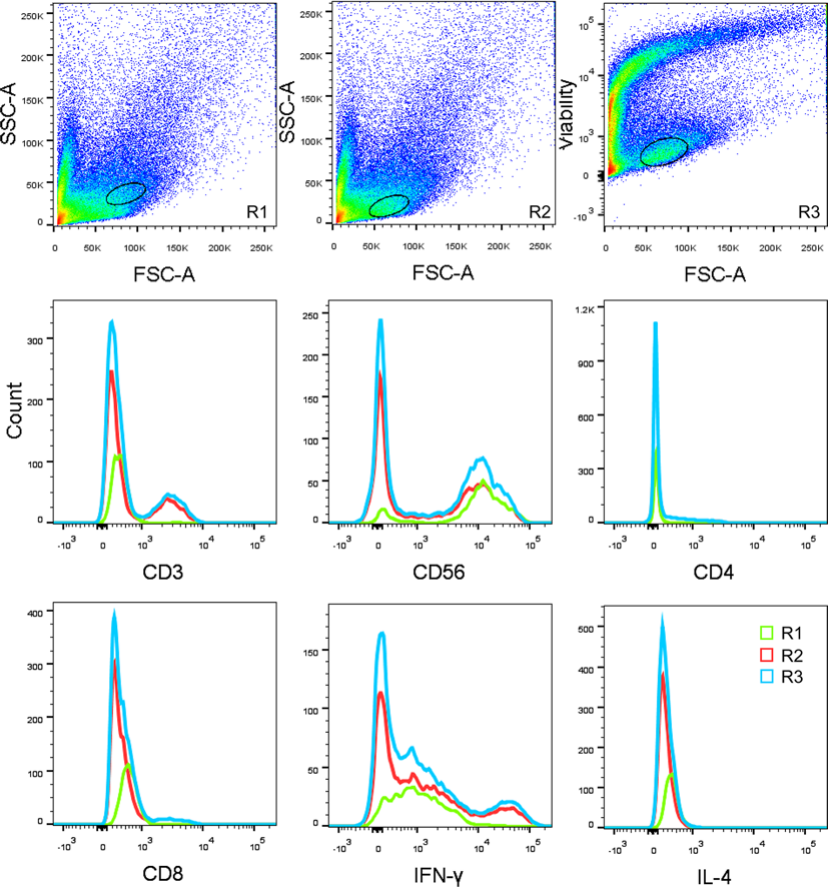
One example to show the advantage of the FSC/Vt gating strategy Three graphs from the same sample from which the dMo were not distinguishable by the FSC/SSC but clustered well by the FSC/Vt are shown. The histograms of the wrong gate location (R1) were compared with the right gate (R2), with the FSC/Vt as a reference (R3). The counts from the R1 are much lower than any other gate for any of the cell markers.

However, the type1/type2 ratios differed significantly only with the FSC/Vt extended strategy. As described above, the reason for this might be that the permeabilising and activating processes alter the SSC parameter. There were rare type1 or type2 subpopulations outside the FSC/SSC gate. Thus, their percents were underestimated, and their ratios were not accurate. In fact, they were much lower while calculating the composition of the parental lineages added together. This was illustrated in the flow chart visually (Figure 1D). The gating by the FSC/Vt extended strategy in the FSC/SSC plot showed that there were a large amount of cells outside the FSC/SSC gate, which may exceed the FSC/SSC extended scale used in the present study. Thus, this may be an advantage of the FSC/Vt extended gating strategy, especially when analyzing rare functional subsets in early decidua. Moreover, we found that the FSC/Vt gate was very useful when the mononuclear cells could not be easily distinguished (Figure 4). Thus, taking advantage of different gating strategies may be of great potential for the accurate identification of much more parameters.

In the present study, we used an FSC/Vt extended gating strategy to simultaneously detect changes in the Th1/Th2 and dNK1/dNK2 ratios in 20 URM cases. We cannot say that the simultaneous detection of the Th1/Th2 and dNK1/dNK2 ratios can be a potential method to become a diagnostic criterion unless we examine these ratios in a larger number of URM cases, which is a limitation of the present study. Thus, using 8-color flow cytometry with an FSC/Vt extended strategy could be a useful means to examine larger sample size investigations in URM in the future.

In Figure 2A, we found that there is a significant difference in the cell proportion of the CD3^+^CD56^–^ group, which represents T cell based on the FSC/SSC (central) gating strategy compared with the FSC/SSC (extend) gating strategy, and a similar phenomenon was observed when using the FSC/Vt (central) gating strategy compared with the FSC/Vt (extended) strategy. These results may show that there are other cells, besides the T cells, in the range delineated by the FSC/SSC (extended) and FSC/Vt (extended) gating strategies. We made a Boolean gate using an extended gate minus a centralised gate and found that the cells inside the extended gate and beside the centralised gate were mainly NK cells. That is to say, in the flow chart in our sample, NK cells are distributed in the periphery of T cells.

Mechanical isolation produces a lower yield of decidual leukocytes than enzymatic digestion but may be desirable for antibodies sensitive to collagenase [30]. Our results validated the lower cell density using only a grinding method. When digesting with 1 mg/ml collagenase for 20 min, for the tissues left on the strainer, the overall yield of mononuclear cells doubled. Although, because of the influence of the enzyme, we preferred to use mechanical isolation only.

## Conclusion

To the best of our knowledge, this is the first study to report the simultaneous detection of decidual Tc, Th, NK, and NKT-like cells with respect to their expression of IFN-γ and IL-4. We provide a framework for the identification of the different immune cell lineages present in the decidua and a means to detect the Th1/Th2 and dNK1/dNK2 ratios simultaneously by using flow cytometry with an FSC/Vt extended strategy, which, in the future, may be used for investigating a large number of URM samples.

## Abbreviations

dMo: decidual mononuclear cell
dNK1: decidual NK1
dNK2: decidual NK2
FSC: forward scatter
FVD: fixable viability dye
IFN: interferon
IL: interleukin
MFC: multiparametric flow cytometry
NK: natural killer
PMT: photomultiplier tube
RM: recurrent spontaneous miscarriage
SSC: side scatter
Th: T helper
URM: unexplained recurrent miscarriage
Vt: viability
